# Ocular manifestations of branchio-oculo-facial syndrome: Report of a novel mutation and review of the literature

**Published:** 2010-05-08

**Authors:** M.S. Al-Dosari, M. Almazyad, L. Al-Ebdi, J.Y. Mohamed, Saad Al-Dahmash, Hassan Al-Dhibi, Eman Al-Kahtani, Shahira Al-Turkmani, Hisham Alkuraya, B.D. Hall, F.S. Alkuraya

**Affiliations:** 1 Department of Genetics, King Faisal Specialist Hospital and Research Center, Riyadh, Saudi Arabia; 2 Department of Pharmacognosy, College of Pharmacy, King Saud University, Riyadh, Saudi Arabia; 3 College of Medicine, King Saud University, Riyadh, Saudi Arabia; 4 Department of Ophthalmology, College of Medicine, King Saud University, Riyadh, Saudi Arabia; 5 Vitreoretinal Division, King Khaled Eye Specialist Hospital, Riyadh, Saudi Arabia; 6 Peadiatric Ophthalmology Division King Khaled Eye Specialist Hospital, Riyadh, Saudi Arabia; 7 Department of Pediatrics, University of Kentucky, Lexington, KY; 8 Department of Pediatrics, King Khalid University Hospital and College of Medicine, Riyadh, Saudi Arabia; 9 Department of Anatomy and Cell Biology, College of Medicine, Alfaisal University, Riyadh, Saudi Arabia

## Abstract

**Purpose:**

To report unusual ocular manifestations of branchio-oculo-facial syndrome (BOFS) caused by a novel mutation in activating enhancer binding protein 2 alpha (*TFAP2A*).

**Methods:**

Full ophthalmological evaluation and direct sequencing of *TFAP2A*.

**Results:**

A 10-year-old girl with unusual ocular manifestations of BOFS such as elliptical shaped microcornea and a novel de novo *TFAP2A* mutation was identified.

**Conclusions:**

This report expands the ocular phenotypic spectrum of BOFS and adds to the small number of reported *TFAP2A* mutations.

## Introduction

In 1987, Fujimoto et al. [1] described an apparently novel syndrome that combines branchial sinus, ocular, and craniofacial anomalies and appropriately named it branchio-oculo-facial syndrome (BOFS OMIM 113620). Branchial sinus defects are usually in the form of cervical/infra-auricular skin defects. Craniofacial defects include pseudo-cleft/cleft lip, broad nasal bridge, and high forehead. Pseudo-cleft is a particularly helpful clinical feature that is highly specific to this syndrome. Ocular features are more diverse and include microphthalmia, lacrimal duct obstruction, and coloboma [2]. Since the original description, 81 cases have been reported, and while the phenotype was believed by many to be quite distinct, others have been more skeptical [3]. In particular, the overlap with branchio-oto-renal syndrome (BORS) has raised doubts that BOFS may in fact be an allelic disorder [3]. Both disorders are autosomal dominant and affect branchial structures, but the two developmental disorders, despite their extreme phenotypic variability, do have quite different predilection to certain organs, such as the kidney in the case of branchio-oto-renal syndrome and the eye in the case of BOFS. However, it was the identification of activating enhancer binding protein 2 alpha (*TFAP2A*) mutations in patients with BOFS that provided the unequivocal proof that BOFS is indeed a distinct clinical entity [4].

Since the original description of *TFAP2A* as the disease gene, very few subsequent reports have been published. In this study, we describe a girl with molecularly confirmed BOFS and who also has unusual ocular manifestations that we believe expand the ocular phenotype of this syndrome.

## Methods

### Human subjects

The patient was recruited with written informed consent (KFSHRC IRB #2070023) and had full ophthalmological and dysmorphological evaluations. The parents were also recruited with written informed consent to determine whether the mutation is familial or de novo. The patient and her parents were recruited at King Khalid University Hospital.

### Mutation analysis

Genomic DNA was extracted from the patient and her parents (5 ml of blood in EDTA from each individual) and kept at 4 degrees until processed for DNA extraction the following day. On the patient’s sample, all *TFAP2A* coding exons as well as their flanking intronic sequences were PCR amplified on MyCycler (Bio-Rad, Hercules, CA) (primer sequences and PCR conditions are available upon request). On the parental samples, a targeted PCR amplification of the mutation-containing fragment was performed. Amplicons were purified then bidirectionally sequenced on an ABI 3730xl DNA Analyzer (Applied Biosystems, Foster City, CA). Sequence analysis was performed using DNA Star package (Lasergene, Madison, WI).

## Results

### Clinical evaluation

The index is a 10-year-old girl who was referred to our clinical genetics service for evaluation because of her craniofacial dysmorphia. She was born at term to a 35-year-old gravida 8 para 7 mother following an uneventful pregnancy. Delivery was spontaneous, vaginal, and uncomplicated. At birth multiple anomalies were noted in the form of a high-arched palate and partial cutis aplasia in the right retroauricular region. Apparent microphthalmia prompted a full ophthalmological evaluation that revealed the additional presence of microcornea, iris and chorioretinal coloboma, inferiorly subluxed lenses with mild cataractous changes on the left, and nasolacrimal duct stenosis. High resolution karyotype was normal 46,XX and subtelomeric fluorescence in situ hybridization (FISH) was normal. Metabolic screening, echocardiogram, and kidney ultrasound were also normal. Magnetic resonance imaging of the eyes performed at the age of 4 years revealed an abnormal configuration of both eye globes, with evidence of a triangular shape of the lenses (which are displaced medially and inferiorly), small coloboma at the posterior aspect of both eye globes, normal symmetric appearance of both optic nerves, and normal extraocular muscles. She had mild speech delay, probably related to mild to moderate hearing loss, but is currently doing well in regular school where she is in an age-matched grade; her IQ was measured to be 85.

**Figure 1:**
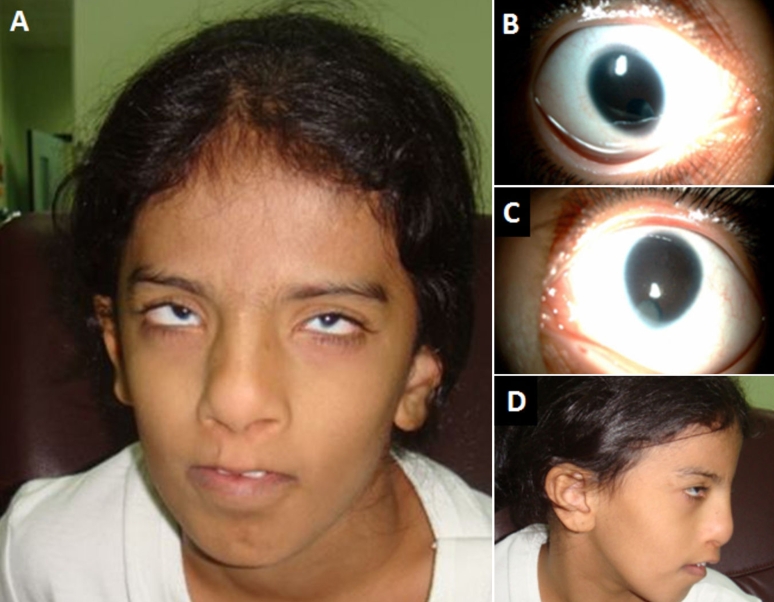
Clinical photographs of the patient. Facial appearance with the typical pseudocleft of the upper lip is shown in **A**. Microcornea, inferonasal coloboma, and cataract are shown in **B** and **C**. Lateral facial profile is shown in **D**.

Physical examination at 10 years of age revealed a small body build, with height at 127.5 cm (between the 5th and 10th centiles), weight at 20.4 kg (<5%), and head circumference at 49.6 cm (just above the 2nd percentile). In addition to the previously documented ocular anomalies, she has pseudocleft of the upper lip, oligodontia, mixed hearing loss, and abnormal orientation of the nasal spine, which appears flushed with the forehead (Figure 1). There is partial cutis aplasia in the right retroauricular region, but the neck appears normal otherwise. The rest of the physical examination was essentially normal. Specifically, there is no evidence of chest, spine, nail, or hair abnormalities. A repeat ophthalmological examination revealed the following:

1- Visual acuity: Searching and wandering eye movement. She did not fix or follow light or objects. Cycloplegic refraction through the phakic part is −1.50 for the right eye and −1.50 for the left eye.
2- Intraocular pressure: Right eye 17 mmHg and left eye 25 mmHg.
3- Slit-lamp examination: Microphthalmia bilaterally, clear but elliptical cornea bilaterally, anterior chamber is deep and quiet, iris shows a large coloboma bilaterally inferiorly with lens opacity. In addition, the lens appeared to be slightly subluxated inferiorly with zonules on both sides showing superiorly.
4- Fundus examination: Showed a large chorioretinal coloboma in both eyes. B-scan ultrasound confirmed a chorioretinal coloboma close to the disc in both eyes, but no retinal detachment or any other pathology was appreciated.

### Mutation analysis

**Figure 2:**
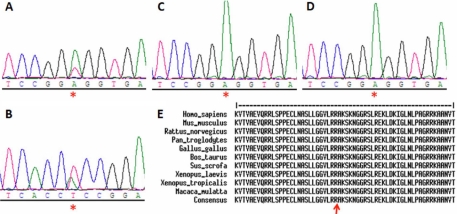
A novel mutation in *TFAP2A*.  Sequence chromatogram of the patient is shown in **A** and **B** (forward and reverse) and that of her parents in **C** and **D** (forward only) with the c.763A>T mutation indicated by a red asterisk.  Protein alignment across species in **E** shows very strong conservation of the R255 residue which is indicated by the red arrow.

**Figure 3:**
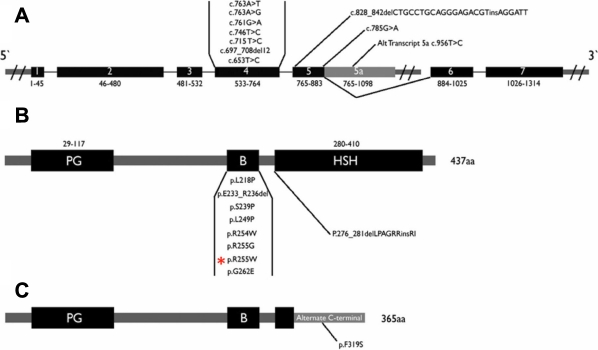
Summary of previously reported *TFAP2A* mutations.  A schematic of the *TFAP2A* gene is shown in **A** along with the genomic location of previously reported mutations. **B** and **C** show two different isoforms of the protein encoded by *TFAP2A* (AP2) along with the location of previously reported mutations at the protein level.  The mutation in the current study is indicated by red asterisk.  PG: proline/glutamine rich domain, B: basic DNA binding domain, HSH: helix span helix domain.

A novel heterozygous *TFAP2A* missense mutation in exon 4, c.763A>T, was detected that results in replacement of basic arginine by nonpolar tryptophan (p.Arg255Trp; Figure 2). This mutation was confirmed on the reverse direction and on repeat PCR on the patient but not on her parents (paternity was verified), thus confirming the de novo nature of this mutation (Figure 2). The mutation affects a highly conserved amino acid residue (Figure 3) and was not found in a panel of 106 Saudi normal controls (212 chromosomes), further supporting its pathogenic nature.

## Discussion

Ocular manifestations of BOFS are variable, as shown in Table 1, with microphthalmia, coloboma, and nasolacrimal duct stenosis being the most common. To our knowledge, the appearance of an elliptical-shaped cornea has never been reported in association with BOFS. Other unusual eye findings in our patient include lens subluxation and microcornea, which have only been observed in three BOFS patients to date (Table 1) [2-21].

**Table 1:** The reported ocular manifestations of BOFS.

	**Reference**																						
**Ocular feature**	**[****2****]**	**[****7****]**	**[****6****]**	**[****5****]**	**[****8****]**	**[****3**]*	**[****9****]**	**[****10****]**	**[****11****]**	**[****14****]**	**[****12****]**	**[****13****]**	**[****15****]**	**[****16****]**	**[****18****]**	**[****19****]**	**[****4**]**	**[****21****]**	**[****20****]**	**[****17****]**	**This study**	**Total**	**%**
Elliptical shaped cornea	0\15	0\2	0\2	0\2	0\2	0\2	0\1	0\1	0\1	0\1	0\1	0\3	0\1	0\1	0\1	0\1	0\5	0\1	0\5	0\5	1\1	1\54	2
Microphthalmia	14\42	1\2	0\2	1\2	1\2	0\2	0\1	1\1	0\1	0\1	1	0\3	0\1	0\1	0\1	1\1	3\5	0\1	2\5	4\5	1\1	30\81	37
Anophthalmia	4\42	0\2	0\2	0\2	0\2	0\2	0\1	0\1	0\1	0\1	0\1	0\3	0\1	0\1	0\1	0\1	0\5	0\1	0/5	0/5	0\1	4\81	4.9
Small PF	9\42	0\2	0\2	0\2	1\2	0\2	0\1	0\1	0\1	0\1	0\1	0\3	0\1	0\1	1\1	0\1	0\5	0\1	0/5	0/5	0\1	11\81	14
NLDS	29\39	2\2	0\2	2\2	2\2	2\2	1\1	0\1	0\1	1\1	1\1	0\3	1\1	1\1	0\1	0\1	2\5	0\1	5\5	1\5	1\1	51\78	65
Coloboma	16\35	1\2	1\2	0\2	0\2	1\2	1\1	0\1	0\1	0\1	1\1	0\3	0\1	0\1	0\1	1\1	3\5	0\1	5\5	1\5	1\1	32\74	43
Sublaxation	0\15	0\2	0\2	0\2	0\2	0\2	0\1	0\1	0\1	0\1	1\1	0\3	0\1	0\1	0\1	0\1	0\5	0\1	0/5	1\5	1\1	3\54	6
Catract	8\33	0\2	0\2	0\2	0\2	1\2	0\1	0\1	0\1	0\1	1\1	0\3	0\1	0\1	0\1	0\1	0\5	0\1	2\5	0/5	1\1	13\72	18
Myopia	9\28	0\2	1\2	0\2	0\2	1\2	1\1	0\1	0\1	0\1	0\1	2\3	0\1	0\1	0\1	0\1	0\5	0\1	0/5	0/5	1\1	15\67	22
Strabismus	11\36	0\2	0\2	0\2	1\2	0\2	0\1	0\1	0\1	0\1	0\1	0\3	0\1	0\1	0\1	0\1	0\5	0\1	3\5	0/5	0\1	15\75	20
Ptosis	10\36	0\2	0\2	0\2	0\2	0\2	0\1	0\1	0\1	1\1	0\1	0\3	0\1	0\1	0\1	0\1	0\5	0\1	0/5	0/5	0\1	11\75	15
Hypertolirsm	3\15	0\2	0\2	0\2	1\2	0\2	0\1	1\1	0\1	0\1	0\1	0\3	0\1	0\1	0\1	0\1	0\5	0\1	0/5	0/5	0\1	5\54	9
Telecanthus	0\15	1\2	1\2	0\2	2\2	0\2	0\1	0\1	0\1	0\1	0\1	3\3	0\1	0\1	1\1	0\1	0\5	0\1	0/5	0/5	0\1	8\81	10
Microcrnea	1\15	0\2	0\2	0\2	0\2	0\2	0\1	0\1	0\1	0\1	0\1	0\3	0\1	0\1	0\1	0\1	0\5	0\1	0/5	1/5	1\1	3\54	6
Dermoid cyst	1\15	0\2	0\2	0\2	0\2	0\2	0\1	0\1	0\1	0\1	0\1	0\3	0\1	0\1	0\1	0\1	0\5	0\1	0/5	0/5	0\1	1\54	2
Eyelid cyst	1\15	0\2	0\2	0\2	0\2	0\2	0\1	0\1	0\1	0\1	0\1	0\3	0\1	0\1	0\1	0\1	0\5	0\1	0/5	0/5	0\1	1\54	2
Primary aphakia	0\15	0\2	0\2	0\2	0\2	0\2	0\1	0\1	0\1	0\1	0\1	0\3	0\1	0\1	0\1	0\1	0\5	0\1	0\5	2\5	0\1	1\54	2

Note that the ratios reflect patients who had the ocular feature to total patients examined. Abbreviations: PF, palpebral fissure ; NLDS, nasolacrimal duct stenosis. The asterisk indicates that seven patients were reported in this study but only two were new. The double asterisk indicates that six patients were reported in this study including one previously reported by Lin et al. [2].

The identification of *TFAP2A* as the disease gene in BOFS provided long sought answers to the ocular phenotype of this syndrome. *TFAP2A* is a retinoic acid responsive gene that encodes activating enhancer-binding protein 2 alpha (AP-2α), a member of the AP-2 family of transcription factors that regulate gene expression during embryogenesis of the eye, ear, face, body wall, limbs, and neural tube [4]. The role of AP-2α in eye development has been established in several studies, perhaps the most compelling of which is the demonstration that *Tfap2a* knockout mouse embryos exhibit grossly abnormal ocular development in the form of anophthalmia, aphakia, absent cornea, coloboma, lens stalk, lack of ciliary body and iris formation, absent eyelids, and ectopic neural retina that replaces part of the retinal pigmented epithelium [22,23].

Mechanistic insight into some of these anomalies comes from a more complicated set of data. For instance, targeted deletion of *Tfap2a* in the murine lens placode reduced cadherin 1 (*Cdh1*) expression and increased epidermal growth factor receptor (*Egfr*) and alpha smooth muscle actin (*α-SMA*) expression, indicating abnormal lens epithelial mesenchymal transformation, which may explain the occurrence of cataract [24]. In another study [25] double heterozygous mice for paired box gene 6 (*Pax6*) and *Tfap2a* showed a more severe ocular phenotype than single heterozygotes in the form of persistent lens stalk or lens protrusion into the cornea, which suggests cooperation between the two transcription factors in lens development. On the other hand, insight into the mechanism of the observed posterior segment defects comes from a zebrafish study that examined how partial abrogation of *Tfap2a* affects the expressivity of ocular phenotypes. Knockdown of either bone morphogenetic protein 4 (*Bmp4*) or transcription factor 7-like 1a (*Tcf7l1a*), which encode a Bmp ligand and a transcriptional effector of Wnt signaling, respectively, do not cause an ocular phenotype. However, partial knockdown of *Tfap2a* in either of these two mutants led to abnormal ocular development in the form of coloboma, anophthalmia, and microphthalmia, thus establishing a genetic interaction between *Tfap2a* and these two genes [17].

In the first *TFAP2A* mutation report by Milunsky et al. [4], a total of five mutations were described, one of which was a large genomic deletion and the remaining four were missense mutations mainly in exon 4. Three subsequent papers described a total of six mutations: one indel, one deletion, one large genomic deletion, and three missense [4,17,20,21] (Figure 3). Mutation analysis of *TFAP2A* in our patient showed a de novo novel mutation in exon 4, a mutation hotspot that encodes the basic region of the DNA-binding domain [4]. Four of the five *TFAP2A* mutations in the original paper by Milunsky et al. [4] were also de novo, and this mutation affects the same residue (R255) described in that paper but results in a different substitution. Both our mutation (R255W) and that by Milunsky et al. [4] (R255G) replace the positively charged arginine with nonpolar amino acids in the DNA-binding domain, which is likely to adversely affect the capacity of TFAP2A to bind DNA. Although no functional validation was performed, the fact that the TFAP2A mutational spectrum is not exclusive to missense mutations is highly suggestive of haploinsufficiency as the most likely mechanism. However, the finding that gain of function N-ethyl-N-nitrosourea-induced mutation in mouse results in a highly similar ocular phenotype to that observed in the *Tfap2a* knockout mouse leaves open the possibility that some missense mutations may in fact mediate their effect in human BOFS patients by increasing *TFAP2A* transcriptional activity [26].

In summary, we add one novel mutation to the allelically heterogeneous disorder of BOFS and describe unusual eye findings that expand the ocular phenotype of this disorder.
